# Synergistic Effects of *Danshen* (*Salvia Miltiorrhizae* Radix et Rhizoma) and *Sanqi* (*Notoginseng* Radix et Rhizoma) Combination in Angiogenesis Behavior in EAhy 926 Cells

**DOI:** 10.3390/medicines4040085

**Published:** 2017-11-21

**Authors:** Xian Zhou, Valentina Razmovski-Naumovski, Antony Kam, Dennis Chang, Chunguang Li, Alan Bensoussan, Kelvin Chan

**Affiliations:** 1 National Institute of Complementary Medicine (NICM), Western Sydney University, Penrith, NSW 2751, Australia; v.naumovski@unsw.edu.au (V.R.-N.); k.antony@ntu.edu.sg (A.K.); d.chang@westernsydney.edu.au (D.C.); c.li@westernsydney.edu.au (C.L.); a.bensoussan@westernsydney.edu.au (A.B.); 2 South Western Sydney Clinical School, UNSW Medicine, University of New South Wales, Kensington 2052, Australia; 3 School of Biological Sciences, Nanyang Technological University, Singapore 637551, Singapore; 4 School of Pharmacy and Biomolecular Sciences, Liverpool John Moores University, Liverpool L3 3AF, UK; 5 Faculty of Sciences, TCM Division, University of Technology Sydney, Ultimo, NSW 2007, Australia

**Keywords:** Danshen-Sanqi, synergy, angiogenesis

## Abstract

**Background:** This study investigated the combination effects of the *Danshen* and *Sanqi* herb pair on angiogenesis in vitro. **Methods:** Nine combination ratios of Danshen-Sanqi extracts (DS-SQ) were screened for their angiogenic effects in the human vascular endothelial EAhy 926 cell line via cell proliferation, cell migration and tube formation activities against the damage to the cells exerted by DL-homocysteine (Hcy) and adenosine (Ado). The type of interaction (synergistic, antagonistic, additive) between *Danshen* and *Sanqi* was analyzed using combination index (CI) and isobologram models. The angiogenic activities of key bioactive compounds from *Danshen* and *Sanqi* were tested in the same models. **Results:** DS-SQ ratios of 2:8 and 3:7 (50–300 µg/mL) potentiated angiogenic synergistic effects (CI < 1) in all three assays. The observed wound healing effects of DS-SQ 2:8 was significantly attenuated by phosphatidylinositol-3 kinases (PI3K), mitogen-activated protein kinase (MEK) and extracellular signal-regulated kinases (ERK) inhibitors which inferred the potential mechanistic pathways. Out of all the tested compounds, Notoginsenoside R1 from Sanqi exhibited the most potent bioactivity in cell proliferation assay. **Conclusions:** This study provides scientific evidence to support the traditional use of the Danshen-Sanqi combination for vascular disease, in particular through their synergistic interactions on previously unexamined angiogenic pathways.

## 1. Introduction

The complexity of vascular diseases such as myocardial infarction and ischemic stroke requires a polypharmacy approach to their treatment. For thousands of years, traditional Chinese medicine (TCM) has used herbs in specific combination ratios to treat the multifactorial nature of disease. Combination therapy is formulated to provide greater therapeutic benefits owing to its enhanced total effects and multi-target behaviors. Synergy is the crucial mechanism in combination therapy aimed to achieve an optimized outcome. Synergy provides a combination greater than that the sum of the individual agents at the same concentration level [1]. In TCM, the result of this synergism is expected to have supplemented and augmented effects, reduced toxicity and enhanced bioavailability [2]. However, there are limited studies that investigate the underlying synergistic effects of herbal formulations in ischaemic vascular diseases [1].

Angiogenesis is an essential physiological process in the vascular system for maintaining blood vessel homeostasis by facilitating nutrient transportation, as well as promoting tissue growth and repair [3]. It is a regenerative response triggered in the injured tissue adjacent to the ischaemic core for rebuilding the blood vessel and delivering the oxygen to distant organs [4,5]. Insufficient angiogenesis can complicate conditions such as myocardial infarction and ischaemic stroke, which are associated with inadequate blood supply due to angiogenic inhibitors or diminished level of vascular endothelial growth factors (VEGF), etc., [6]. Presently, therapeutic angiogenesis is considered as a novel approach for improving the outcome of ischaemic vascular diseases [7]. Here, the therapeutic goal is to enhance perfusion, deliver survival factors to sites of tissue repair, mobilize regenerative stem cell populations and ultimately restore form and function to the tissue [8].

The herbal combination of *Danshen* [*Salvia Miltiorrhizae* Radix & Rhizoma; Lamiaceae] and *Sanqi* [*Notoginseng* Radix et Rhizoma; Araliaceae] is a popular formula widely used in Asian countries traditionally for promoting blood circulation and wound healing [9,10]. A generic formulation listed in the official Pharmacopoeia of the People’s Republic of China, the Fufang Danshen tablet/pill/granule (consisting *Danshen* 450 g, *Sanqi* 141 g and Borneol 8 g), is indicated for angina pectoris in coronary heart disease [11]. Clinical studies have shown positive outcomes of the combination in improving vascular-related conditions such as chronic angina, diabetic retinopathy and cervical intraepithelial neoplasia [12,13,14]. Modern pharmacological studies have shown that *Danshen* causes relaxation of the coronary arteries which is one of the herb’s major physiologic, cardioprotective effects, whereas *Sanqi* serves as the deputy herb in the formulation because of its cardiomyocyte protective and antiplatelet function [15,16,17].

In particular, numerous pharmacological studies have reported the angiogenic activities of *Danshen* and *Sanqi* as a single extract [18,19,20]. For example, phenolic acid constituents (i.e., sodium danshensu, salvianolic acid B) from *Danshen* were shown to be active in promoting blood vessel growth [9]. Total saponins from *Sanqi* were shown to stimulate cell proliferation, migration and tube formation in HUVEC cells [10]. One of its major active chemical components—ginsenoside Rg1 (Rg1)—is considered to be a potent pro-angiogenic agent in vitro (HUVEC) and in vivo (zebra fish model) [21]. PI3k/Akt (protein kinase B) signaling pathway was associated with Rg1-induced angiogenesis [10,22]. However, efficacy studies on the combined angiogenic activity of *Danshen* and *Sanqi* are lacking.

In this study, the activities of the aqueous extracts of these herbs and combination were investigated using three in vitro angiogenesis bioassays: endothelial cell proliferation, endothelial cell migration and tube formation. Furthermore, the combination activity of a Danshen-Sanqi extract combination was analyzed using mathematical combination index (CI) and isobologram model to investigate whether the action of the herbal combination could be explained by synergism, addition or antagonism [23]. A mechanistic explanation using the associated mechanical pathways pertaining to PI3K, MAPK, ERK and ENOS was proposed. In addition, the underlying mechanistic behavior of the synergistic interactions were further investigated by the evaluating the associated activities of purified major bioactives of these herbs.

## 2. Materials and Methods

### 2.1. Cell Line and Culture Conditions

Human cardiovascular endothelial cell line (EAhy926) was provided by Monash University Central Clinical School, Australia. The cell line was cultured in DMEM/Ham’s F12 containing 15 mM HEPES and L-glutamine and supplemented with 10% FBS, 100 U/mL of penicillin and streptomycin (Gibco BRL, Scoresby, Australia). The cell line was grown in a 5% CO_2_-humidified incubator at 37 °C.

### 2.2. Preparation of Herbal Samples and Their Chemical Compounds

Crude *Danshen* and *Sanqi* herbal materials were sourced from PuraPharm International Ltd., Hong Kong. The raw herbal materials were authenticated by Professor Si-bao Chen from the Department of Applied Biology and Chemical Technology, the Hong Kong Polytechnic University, Hong Kong, China, according to the Hong Kong Materia Medical Standards. The aqueous extracts of *Danshen* (DS) and *Sanqi* (SQ) were prepared as follows: 1 g of Danshen/Sanqi crude herbal material was ground to a powder and sieved through a 30-meshsize to obtain a fine powder. The powder was weighed and soaked in 30 mL hot water for 0.5 h, followed by refluxing with boiling water for another 1 h. This step mimics the traditional way of making herbal decoction. The “decoction” was then centrifuged at 3000 rpm for 5 min. The supernatant was separated, collected and evaporated to dryness using a freeze dryer and stored at 4 °C for further use. For the preparation of the combined aqueous extracts of *Danshen* and *Sanqi* (DS-SQ), the crude herbal powder of *Danshen* and *Sanqi* was combined in nine different (*w*/*w*) ratios (1:9, 2:8, 3:7, ..., 8:2, 9:1) and extraction proceeded as for the single extract. Chemical standards for Danshen including sodium danshensu (DSS), salvianolic acid B (SB), salvianolic acid A (SA), tanshinone TIIA (TIIA), dihydrotanshinone I (DT), tanshinone I (TI) and cryptotanshinone (CT) and those for Sanqi including ginsenoside Rg1 (Rg1), ginsenoside Rg2 (Rg2), ginsenoside Rd (Rd), ginsenoside Rb1 (Rb1) and notoginsenoside R1 (NR1) were purchased from Chengdu Biopurify Phytochemicals Ltd. (Chengdu, China; purity > 98%). The chemical fingerprint of DS and SQ are shown in Figure S1. The content of chemical standards in both single and combined extracts of DS and SQ is shown in Figure S2. The standard stock solutions of these reference compounds were prepared in methanol and stored at 4 °C until required.

### 2.3. Cell Proliferation Assay

EAhy 926 cells (1.0 × 10^4^/well) were seeded in a 96-well plate for 2 h prior to the co-incubation of Hcy, Ado and/or herbal extracts (final dimethyl sulfoxide (DMSO) concentration 0.1%) in a serum-free medium. After a 72-h incubation period, 3% buffered paraformaldehyde was added to the cell supernatant for 20 min. The cells were then stained with 0.2% crystal violet in 20% methanol for 5 min. The excess crystal violet stain was rinsed 4–5 times with distilled water and air-dried. Glacial acetic acid (33%) in Milli-Q water was added to remove the crystal violet stain. Finally, the optical density was measured using a microplate reader (BMG Labtech Fluostar Optima, Mount Eliza, Victoria, Australia) at the wavelength of 595 nm (Martin & Clynes, 1993).

### 2.4. Cell Migration (Scratch Wound Healing) Assay

A wound scratch assay was performed according to Gebäck’s protocol [24]. Briefly, 1 × 10^4^ EAhy926 cells per well were seeded on a 24-well plate and grown to confluence. The cell monolayer was then scratched using a 1000 μL pipette tip and rinsed gently with phosphate-buffered saline (PBS) to remove cell debris. Two cross-shaped gaps were scratched in each well and these were instantly center-imaged at 4× magnification, using a Motic AE20 microscope and a Tucsen ISH500 digital camera with maximum contrast. The photo was viewed and recorded with ISCapture software, labelled as 0 h and the cells were exposed to Hcy, Ado and/or sample treatments in serum-free medium. After 24 h incubation, the wound in the same well was photographed again and recorded as 24 h. The percentages of cell-free areas were then compared between 0 and 24 h and the data was analyzed using TScratch software [24].

### 2.5. Tube Formation Assay

Tube formation assay was performed according to Arnaoutova & Kleinman (2010). Briefly, 50 μL Cultrex Basement Membrane Extract per well was used to coat a 96-well flat-bottom plate. The plate was centrifuged at 250× *g* for 10 min. The plate was then incubated at 37 °C for at least 30 min to initiate gelling. EAhy926 cells (1 × 10^4^) were then seeded in each well and incubated for 2 h. After incubation, the cells were treated with Hcy, Ado and/or herb treatments in serum-free medium. After 20 h of incubation, the well was photographed using the inverted phase-contrast microscope. The total number of junctions (consisting of at least three branches) was quantified using a plug-in, Angiogenesis Analyzer in NIH Image J software (National Institutes of Mental Health, Bethesda, MD, USA).

### 2.6. Determination of Synergistic, Additive, or Antagonistic Interactions

The potential interactions of the combined extract in each assay were analyzed using the CI and isobologram model [25]. The concentration-response curves of the individual extract/compounds and their combinations (in a fixed ratio) pertaining to the bioassay were constructed and the data was entered into CompuSyn software 2.0 (Biosoft, Cambridge, UK). The graphs of combination index-fraction affected (CI-Fa) curve, isobologram and the relevant statistics of the synergistic/additive/antagonistic interactions were generated. The CI-Fa curve demonstrated the correlation between the interactions and the effective level for a certain biological target for each assay (i.e., synergistic interaction at the 90% promoted cell proliferation level). The CI values denoted synergism (CI < 1), additive effect (CI = 1) and antagonism (CI > 1). This curve exhibited the synergistic/antagonistic interaction at an effective range.

### 2.7. Mechanistic Pathways

The possible involvement of the PI3K, eNOS, MEK and ERK pathways were investigated using the relevant inhibitors, including wortmannin (a PI3K inhibitor, at 2 μM), L-NIO dihydrochloride (eNOS inhibitor, at 100 μM), U0126 (MEK inhibitor, 20 μM) and PD98059 (a specific inhibitor of ERK1/2, 2 μM) on cell migration assay. Each inhibitor was co-incubated with the analyte in the cell line to examine whether the wound healing effect of the analyte was affected by any of the inhibitors.

### 2.8. Statistical Analysis

Statistical comparisons were performed using GraphPad Version 5.02 (GraphPad Software, Inc., La Jolla, CA, USA). The data was analyzed by one-way analysis of variance (ANOVA). Data was expressed as mean ± SEM. *p* < 0.05 was considered as statistically significant.

## 3. Results

### 3.1. Effects of DS, SQ and DS-SQ on Hcy-Mediated Angiogenesis in EAhy 926 Cells

#### 3.1.1. Effects of DS, SQ and DS-SQ on Cell Growth

**Figure 1:**
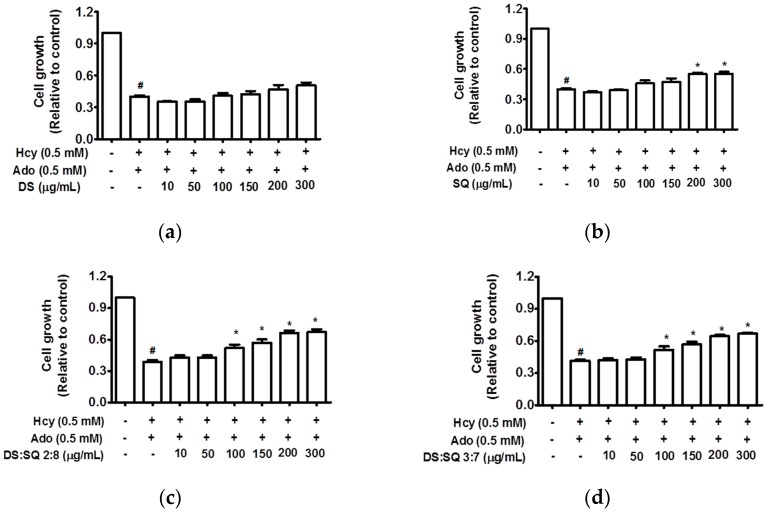
Cell growth (relative to control) concentration curves for DS (**a**), SQ (**b**), DS-SQ 2:8 (**c**) and DS-SQ 3:7 (**d**) aqueous extracts on cell proliferation assay. ^#^ *p* < 0.05 compared with blank control (no Hcy or Ado); * *p* < 0.05 compared with Hcy and Ado stimulation.

Hcy and Ado caused a time- and concentration-dependent reduction of cell proliferation without affecting cell viability. It is observed that the growth of EAhy 926 cells was significantly attenuated by both Hcy and Ado (0.1–0.5 mM) (*p* < 0.05) (Figure 1) and the greatest inhibition (40.2 ± 4.9%) was observed at the 72-h incubation time point.

DS extract did not show any significant improvements (*p* > 0.05), however, SQ extract significantly restored cell growth at concentrations greater than 200 μg/mL (*p* < 0.05). The combination extracts of DS and SQ at all nine ratios showed significant improvements on impaired cell growth in a concentration-dependent manner. Among all the combinations, DS-SQ ratios of 2:8 and 3:7 demonstrated prominent protective effects when used at a dose range of 100 μg/mL and above (*p* < 0.05) (Figure 1), whereas other ratios showed significant effects with higher concentrations at 200–300 μg/mL (*p* < 0.05) (data not showed).

#### 3.1.2. Effects of DS, SQ and DS-SQ on Wound Healing

**Figure 2:**
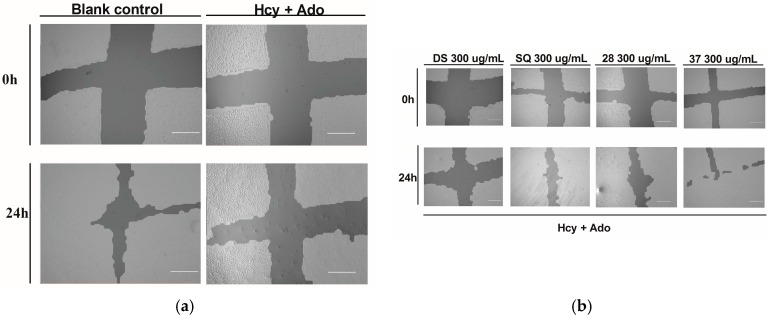

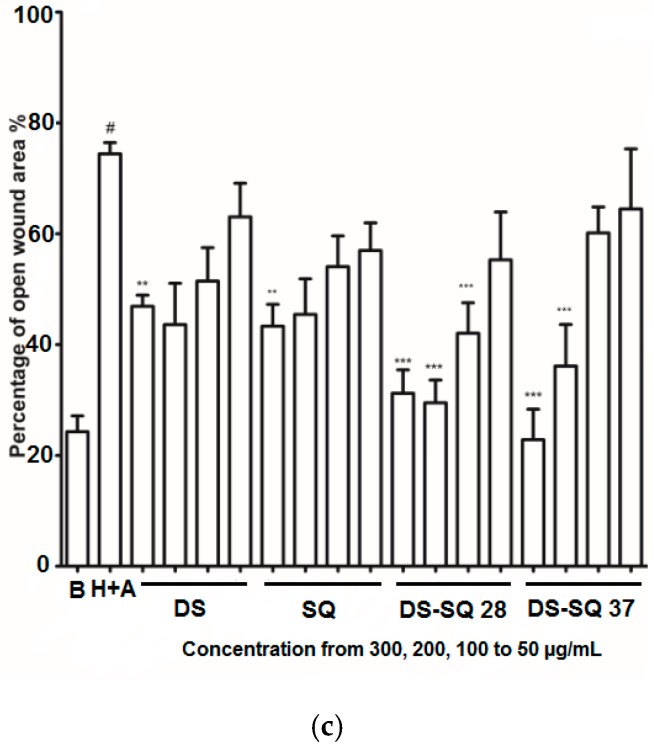
DS, SQ and DS-SQ on EAhy 926 endothelial cell wound scratch assay. The photos of EAhy926 cells with treatment of DS, SQ, DS-SQ 2:8 or DS-SQ 3:7 after wounding at 0 h and 24 h were taken and segmented automatically by TScratch, with identical settings for bright-field acquired. The percentage of the open wound area for each sample generated by TScratch was recorded and the percentage of open wound area was calculated by: $\frac{\mathrm{the}\;\mathrm{percentage}\;\mathrm{of}\;\mathrm{open}\;\mathrm{area}\;\mathrm{at}\;24\;h\times 100\%}{\mathrm{the}\;\mathrm{percentage}\;\mathrm{of}\;\mathrm{open}\;\mathrm{area}\;\mathrm{at}\;0\;h}$ (**a**) EAhy 926 cells with medium only after wounding at 0 h (top-left) and 24 h (bottom-left) and the cells treated with Hcy and Ado at 0 h (top-right) and 24 h (bottom-right); (**b**) EAhy 926 cells co-incubated with DS, SQ, DS-SQ 2:8 and DS-SQ 3:7 at 300 µg/mL after wounding at 0 h (top) and 24 h (bottom) and Hcy-Ado; (**c**) Statistical analysis for DS, SQ and DS-SQ 2:8 and 3:7 on wound healing activities in EAhy 926 cells induced by Hcy and Ado. “H+A” represents Hcy and Ado stimulation. All results were expressed as mean ± S.E.M. from three separate experiments. ^#^ *p* < 0.05 compared with blank. * *p* < 0.05 compared to Hcy plus Ado treated group. ** *p* < 0.01 compared to Hcy plus Ado treated group. *** *p* < 0.001 compared to Hcy plus Ado treated group.

As shown in Figure 2a,c, the open wound area of cells without Hcy and Ado stimulation became significantly smaller after 24 h of incubation due to cell growth and migration. Conversely, treatment with Hcy and Ado significantly inhibited the scratch wound closure within a 24 h period (*p* < 0.05).

DS, SQ, DS-SQ (2:8) and DS-SQ (3:7) all significantly restored the impaired wound healing activity against Hcy and Ado in a concentration-dependent manner (Figure 2c). The wound healing effects of DS and SQ alone were significant (*p* < 0.05) when concentration reached 300 µg/mL (Figure 2b,c). In particular, DS-SQ (3:7) and DS-SQ (2:8) showed significant wound healing effects (*p* < 0.05) at 200 µg/mL and 100 µg/mL, respectively.

#### 3.1.3. Effects of DS-SQ on Tube Formation

**Figure 3:**
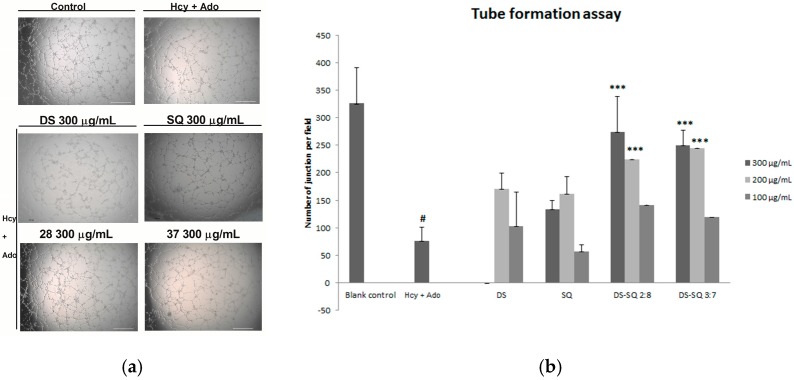
Effects of DS, SQ, DS-SQ 2:8 and DS-SQ 3:7 on angiogenic activities in EAhy 926 cells induced by Hcy and Ado. (**a**) Photo view of blank, Hcy + Ado and DS, SQ, DS:SQ 2:8 and DS:SQ 3:7 at 300 µg/mL on tube formation in EAhy 926 cells; (**b**) Statistical analysis of number of junction per field for each sample, generated by angiogenesis analyzer, Image J. All results were expressed as mean ± SD from over three independent experiments. ^#^ *p* < 0.05 Hcy + Ado stimulation compared with blank control. * *p* < 0.05 compared with Hcy + Ado stimulation. *** *p* < 0.001 compared with Hcy + Ado stimulation.

After 24 h incubation, many tube-like structures were observed on the matrigel, as shown in Figure 3. Hcy and Ado significantly reduced the tube-like structures and junctions of cells compared to the control (*p* < 0.05), indicating its suppressing effects on tube formation (Figure 3a).

When the EAhy926 cells were treated simultaneously with 50 to 300 µg/mL of DS, no significant improvement was observed (*p* > 0.05). Similarly, SQ did not show any significant effects compared to the Hcy and Ado group at all tested concentrations (50–300 µg/mL). In contract, DS-SQ 2:8 and 3:7 at 200 and 300 µg/mL significantly restored tube formation impaired by Hcy and Ado (Figure 3b).

#### 3.1.4. Synergy Analysis of Combination Effects of DS-SQ on Cell Proliferation, Wound Healing and Tube Formation

**Figure 4:**
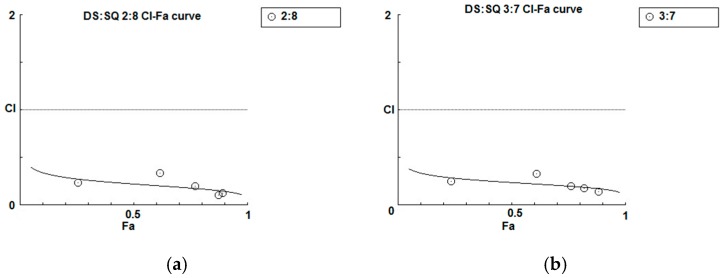

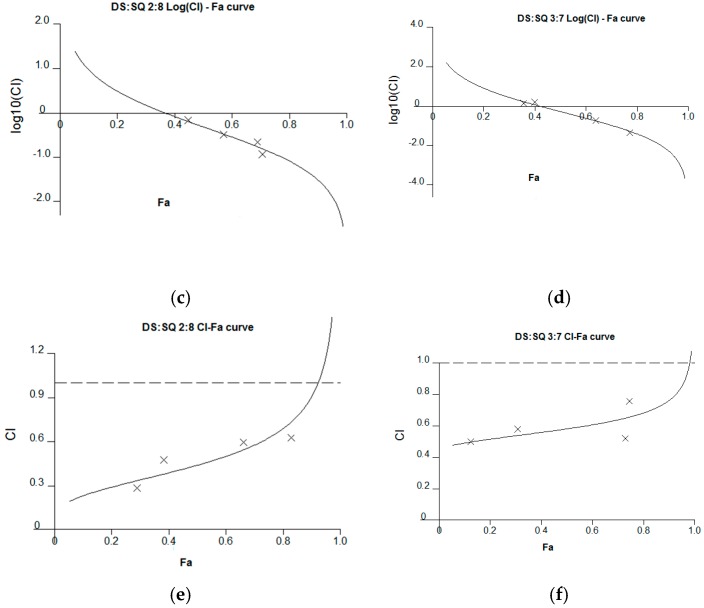
Synergism analysis of DS-SQ 2:8 and 3:7 on restoring cell proliferation, wound scratch and tube formation activities against Hcy and Ado in EAhy 926 cells. CI-Fa curves for DS-SQ 2:8 (**a**) and DS-SQ 3:7; (**b**) on cell proliferation. Log (CI)-Fa curves for DS-SQ 2:8; (**c**) and DS-SQ 3:7; (**d**) on wound healing. CI-Fa curves for DS-SQ 2:8; (**e**) and DS-SQ 3:7; (**f**) on tube formation. CI values were plotted as a function of cell wound healing activity (Fa) by ‘Calcusyn’ software. Solid line is the reference line, where Log(CI) value equals 0 (CI = 1). The curve with spots represents CI values at different Fa. Isobolograms of DS-SQ 2:8 (E) and DS-SQ 3:7 (F) at different effect levels (50%, 75% and 90%).

As shown in the concentration-effect curves of cell proliferation activity of DS, SQ, DS-SQ 2:8 and DS-SQ 3:7 (Figure 1), stronger effects (Fa value) of the 2:8 and 3:7 combinations were observed, compared to DS and SQ alone at all tested concentrations. The CI-Fa curves in Figure 4a,b suggested that the CI values were lower than 1 at all Fa values (at all tested concentrations) for DS-SQ 2:8 and 3:7.

For the wound healing activity, the stronger effects of the 2:8 and 3:7 combinations were observed, compared to DS and SQ alone, at 200–300 µg/mL (Figure 2). A general synergistic effect [Log (CI) < 0] was also observed for the 2:8 and 3:7 combinations when the wound healing effect level (Fa) was higher than 38% and 41%, respectively (Figure 4c,d). From the calculations, there was synergistic-enhanced activity for wound healing in 2:8 and 3:7 when the concentrations for the combination were greater than 50 and 100 µg/mL, respectively.

For the tube formation assay, the combined activity of DS-SQ 2:8 and 3:7 on tube junction number relative to control were constantly higher than the single extract at all tested concentrations (Figure 3). Moreover, the CI model revealed that the combined effects of 2:8 and 3:7 were synergistically enhanced at all tested concentrations (Figure 4e,f).

#### 3.1.5. Mechanistic Pathways for DS-SQ on Hcy-Mediated Angiogenesis in EAhy 926 Cells

**Figure 5:**
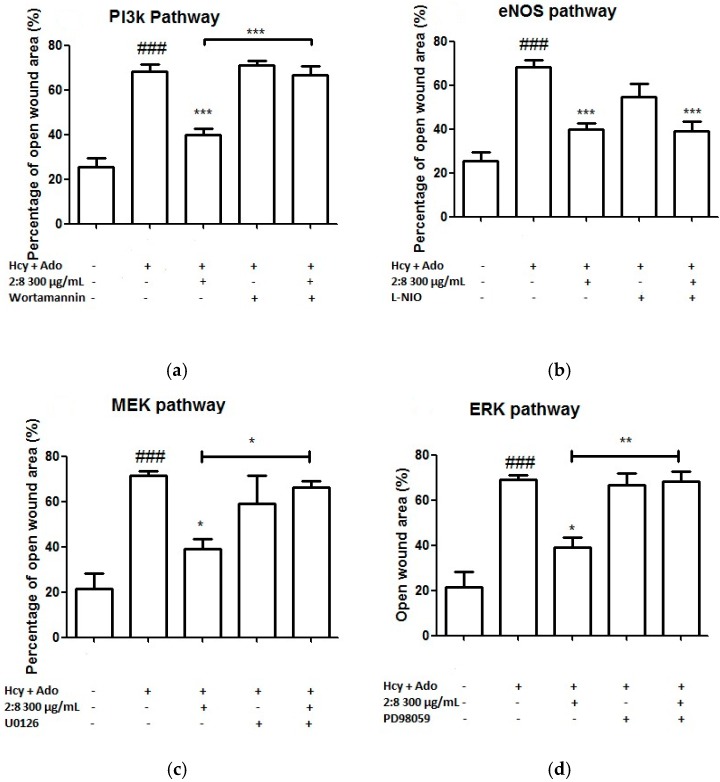
Effects of (**a**) wortmannin; (**b**) L-NIO dihydrochloride; (**c**) U0126 and (**d**) PD 98059 on EAhy926 cell growth affected by Hcy and Ado. EAhy926 cells were exposed to Hcy, Ado, DS-SQ 2:8 and/or each inhibitor for 24 h in serum-free medium after wound scratch. The open wound area before and after the treatment was analysed by TScratch software. The percentage of open wound area was calculated by: the percentage of open area at 24 h/the percentage of open area at 0 h × 100%. All results were expressed as mean ± S.E.M. from three separate experiments in triplicate. ^#^ *p* < 0.05 compared to control. * *p* < 0.05 compared to Hcy plus Ado treated group. ** *p* < 0.01 compared to Hcy and Ado treated group.

To investigate the likely associated signalling pathways (including PI3K, eNOS, ERK/MEK) of the combined effects of DS-SQ on angiogenesis against Hcy and Ado in vitro, the inhibitor of each associated protein was added to the wound scratch assay and the results were analysed. As shown in Figure 5, wortmannin (a PI3K inhibitor), L-NIO dihydrochloride (eNOS inhibitor), PD98059 (a specific inhibitor of ERK1/2) or U0126 (MEK inhibitor) alone did not show any significant impairment or promotion of wound healing in the EAhy 926 cells (*p* > 0.05). However, the co-incubation of wortmannin, U0126 and PD98059 significantly reversed the wound healing activity of the DS-SQ 2:8 at 300 µg/mL (Figure 5a,c,d). Only L-NIO dihydrochloride did not affect the wound closure activity of DS-SQ 2:8 (Figure 5b). Thus, this suggests that the angiogenic activity of DS-SQ 2:8 against Hcy and Ado is associated with the PI3K and ERK/MEK signalling pathway.

#### 3.1.6. Angiogenic Effects of Chemical Compounds from DS and SQ on Hcy-Mediated Angiogenesis on EAhy 926 Cells

**Figure 6:**
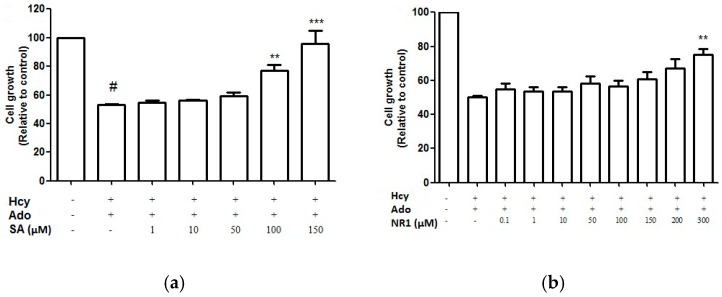
Cell growth (relative to control)-dose-response curves for SA (**a**) and NR1 (**b**) from DS and SQ, respectively. ^#^ *p* < 0.05 compared to blank control, * *p* < 0.05 compared to Hcy-Ado stimulation only.

Fourteen key bioactive compounds from DS—Including DSS, SA, SB, CT, TI, DT, TIIA and SQ—NR1, Rg1, Re, Rb1, Rb2, Rd and Rg2 were screened for cell proliferation activity using the crystal violet staining assay. SA and NR1 were found to improve endothelial cell proliferation in a concentration-dependent manner (Figure 6) and significant improvement was shown at above 100 μM and 300 μM, respectively (*p* < 0.05). However, when SA and NR1 were further tested on wound scratch and tube formation assay, SA and NR1 did not show any significant effects at all tested concentrations (*p* > 0.05).

## 4. Discussion

Angiogenesis is a regenerative response triggered in the injured tissue adjacent to the ischaemic core for rebuilding the blood vessel and delivering the oxygen to distant organs [4,5]. The three models of cell proliferation, cell migration and tube formation established in this study were based on Hcy-impaired angiogenic pathways. In recent years, there is increasing evidence to suggest the importance of Hcy as a new and important risk factor/independent predictor for vascular diseases such as ischaemic heart diseases and stroke. An accumulation of Hcy, often referred to as hyperhomocysteinaemia, has been shown to concentration-dependently impair endothelial cell proliferation and survival together with Ado and ultimately lead to vascular dysfunction [25,26,27]. It is suggested that increased extracellular level of hyperhomocysteinaemia in response to cell injury and stress (i.e., ischemia) with Ado [28,29] produces elevated extracellular S-adenosylhomocysteine which in turn, reduces the intracellular mRNA and protein expressions of fibroblast growth factor (FGF2) via G-protein receptor thus, leading to endothelial anti-proliferation (Hcy = 50–100 µM) and anti-survival activity (Hcy > 100 µM) [30,31,32].

Consistent with the previous findings [27,33,34], we have successfully established the Hcy-impaired cell proliferation, migration and tube formation model on EAhy 926 cells. Our results showed that Hcy and Ado impaired angiogenesis in these three assays in a concentration and time-dependent manner. The concentration of Hcy and Ado at both 0.5 mM significantly and consistently attenuated the cell growth, migration and tube formation of EAhy 926 cells throughout the experiments in comparison to the blank control (*p* < 0.05), however, it did not impact the cell viability. Such impairment of Hcy on angiogenesis was shown to be associated with PI3K, MEK and ERK pathways in our study [32].

In Chinese medicine, the herbs are usually used in combination to enhance synergistic activity. Therefore, we have demonstrated the combined activities of *Danshen* and *Sanqi* water extracts in comparison to the single extracts on three in vitro angiogenesis processes including cell proliferation, cell migration and tube formation and supports the traditional, combined use of herbs in TCM. To the best of our knowledge, this is the first report of the synergistic effects of a DS-SQ combination, particularly on the Hcy- mediated angiogenetic pathway associated with vascular diseases. In our results, the synergistic effects of the DS-SQ combinations were observed for all three bioassays. Our results revealed that the DS-SQ paired herb at all tested ratios (1:9 to 9:1) synergistically restored inhibited cell growth against Hcy and Ado and the effects were more prominent than its single counterpart. Among them, the DS-SQ ratios of 2:8 and 3:7 were found to be the optimal ratios for their potent protective effects. The synergistic interactions were confirmed by CI model at all tested dosage (50–300 μg/mL). In addition, DS-SQ 2:8 and 3:7 consistently restored the scratch wound and tube formation against Hcy and Ado. This finding suggested, for the first time, that there are synergistic interactions between *Danshen* and *Sanqi* in the mixed combination for promoting Hcy-mediated angiogenesis on EAhy 926 cells. In comparison, DS alone did not exert any significant angiogenic activity against Hcy and Ado-induced impairment, with only a slight wound healing effect at 300 µg/mL. On the contrary, SQ significantly promoted angiogenesis against Hcy and Ado via restoring cell proliferation and wound healing at 200–300 µg/mL and 300 µg/mL, respectively, which the effective concentration range was still higher than that of the combination.

It is suggested that the underlying mechanisms of the observed synergistically-enhanced bioactivity is associated with the increased extraction yield of chemical compounds, physicochemical reactions among compounds, multi-target behavior or enhanced bioavailability [35]. For the chemical composition of the DS-SQ combination, previous studies using ultraviolet-visible spectrophotometry, infrared spectroscopy, high performance liquid chromatography and time-of-flight mass spectrometry indicated that there were new chemical substances formed in the aqueous co-decoction of DS and SQ [36]. A study showed that the extraction yield of chemical constituents from DS was increased when it was co-decocted with SQ and this was related to extraction influences including pH, solvent system and chemical component interactions [37]. In particular, DS-SQ 5:3 yielded the highest number of chemical constituents from DS among all tested ratios [37]. However, none of the studies have identified any newly-formed compounds in the DS-SQ mixture, given the general findings that the DS-SQ mixed preparation was not simply the sum of chemical constituents from the respective single herb. Our ultra-performance liquid chromatography chemical analysis for the combination revealed that the number of chemical compounds (DSS, SB, NR1, Rg1 and Rb1) was not directly proportional to the ratio of their herbal ingredients in the combination (Supporting Material 2). This finding corresponded with Zeng’s et al., (2014) study that the co-decoction gave a higher extraction yield for DS. Therefore, such observed, altered compound compositions in the formula may shed a light on the possible explanations of the observed synergy for this and other herbal combinations.

Moreover, this synergistic effect is concentration and ratio dependent. It is noted that the optimal ratio found in our study is different to the ratio in the PPRC (DS:SQ of 3:1) as suggested for angina pectoris. Thus, this optimal ratio cannot be applied to all targeted events. We have previously revealed that the optimal ratio of DS-SQ in targeting anti-inflammation was 8:2 on RAW264.7 cells, which is different from the findings for angiogenesis in this study [38]. A study by Cheung et al., (2012) showed that the herb pair of Danshen-Gegen (*Pueraria lobata*; Fabaceae) in 7:3 (*w*/*w*) did not necessarily generate synergistic effects in all atherogenic events (synergy in anti-inflammation, addition in foam cell formation and antagonism in vSMC anti-proliferation) [39]. Therefore, the ratio of herbs used in combination will need to be tailored to the desired synergistic effect/outcome. Indeed, this finding has been explained in a TCM classic book for the DS-SQ combination: the larger proportion of Sanqi should be adopted in this herb pair for recovery at the later stage of the vascular disease when the pathological damage of the organs is observed [2]. Since therapeutic angiogenesis is targeted for the recovery of the tissue and blood vessel, our findings of using a higher proportion of SQ in the ratio corresponds to the traditional theory.

Although partially demonstrated, our results suggested that DS-SQ 2:8 was associated with PI3K, MEK and ERK, but not eNOS mechanistic pathways. PI3K, MEK and ERK are the downstream targets for bFGF mediated angiogenesis of which bFGF is the target of Hcy, whereas eNOS is the end target of VEGF-2 mediated angiogenesis. Therefore, we can presume that the observed angiogenic effects of DS-SQ was due to the up-regulated activity on bFGF which protected against Hcy. Further investigation on protein and gene expressions of bFGF and its downstream targets are needed to confirm this assumption.

DS, as a single extract, has been shown to be active in promoting angiogenesis on endothelial cells, with phenolic acids as the major contributor acting via VEGF pathway [9]. Our results suggested that SB was not capable of promoting angiogenesis against Hcy and Ado with any significance. Thus, it is presumed that the angiogenic activity of DS and SB was not associated with Hcy-bFGF-mediated pathway. A previous study has shown that the in vitro and in vivo angiogenic effect of SQ was largely derived from its saponin component—Rg1 [10]. The angiogenic activities of both SQ extract and Rg1 was suggested to be associated with VEFG mediated pathway. In our study, NR1, but not Rg1, showed significant activity in restoring impaired cell proliferation against Hcy at 300 µM. Therefore, the cell proliferation effect of SQ against Hcy was likely to be attributed to NR1. Since SQ showed angiogenic activity to some extent, it was assumed that the other existing compounds together with NR1 exerted enhanced effects for SQ on Hcy-mediated angiogenesis.

Furthermore, our results showed NR1 was the only active compound in promoting cell growth at a high concentration (300 μM) among all tested fourteen compounds of DS and SQ. This is surprising since the DS-SQ combination exerted concentration-dependent cell regrowth. There are several possibilities to explain these results. Firstly, the compounds investigated may not be the active compounds responsible for the actions of DS, SQ and DS-SQ in these particular assays. Secondly, the actions of DS, SQ and DS-SQ may result from the combined actions of many constituents, rather than one compound. Further studies are necessary to elucidate the exact mechanisms involved.

## 5. Conclusions

The findings from the present study demonstrated that the combination of DS-SQ at the ratios of 2:8 and 3:7 produced a significant synergistic effect in promoting endothelial cell growth, scratch wound closure and tube formation in EAhy 926 cells. This effect, which supports TCM combination herb theory and practice, may be related to the actions of the compounds in the combined extract on specific cellular signaling pathways, although exact mechanism needs to be elucidated. Further studies may reveal important molecular mechanisms involved in the actions of DS-SQ and other traditional herbal combinations for evidence-based therapeutic applications.

## Supplementary Materials

The following are available online at www.mdpi.com/2305-6320/4/4/85/s1, Figure S1: Chemical fingerprint of raw herb extract of DS (**A**) and SQ (**B**) analyzed by UPLC-PDA. (1) NR1, (2) Rg1, (3) Rb1, (4) Rg2, (5) Rd, (6) DSS, (7) SB, (8) SA, (9) DT, (10) CT, (11) T1 and (12) TIIA [40], Figure S2: Contents (mg/g, mean ± SD, *n* = 3) of DSS, SB, NR1, Rg1 and Rb1 in DS-SQ combinations extract by UPLC-PDA.
