# Supplementary material for: Synergistic Effects of Danshen (Salvia Miltiorrhizae Radix et Rhizoma) and Sanqi (Notoginseng Radix et Rhizoma) Combination in Angiogenesis Behavior in EAhy 926 Cells
Source: Medicines (Basel). 2017 Nov 21;4(4):85. doi: 10.3390/medicines4040085 (PMC5750609; doi:10.3390/medicines4040085)
Supplement: Supplementary file 1 [file medicines-04-00085-s001.pdf]

Supplementary Materials: Synergistic Effects of *Danshen* (*Salvia Miltiorrhizae* Radix et Rhizoma) and *Sanqi* (*Notoginseng* Radix et Rhizoma) Combination in Angiogenesis Behavior in EAhy 926 Cells

Xian Zhou, Valentina Razmovski-Naumovski, Antony Kam, Dennis Chang, Chunguang Li, Alan Bensoussan and Kelvin Chan

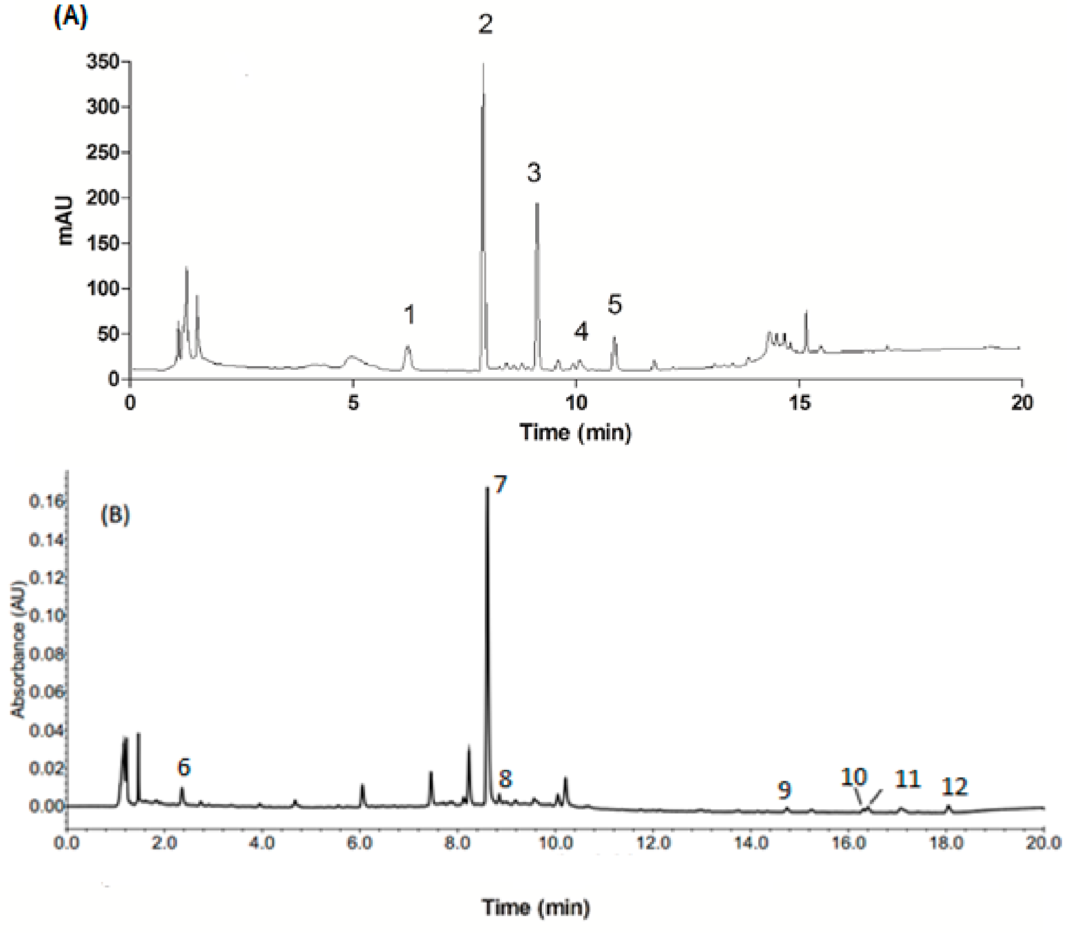

Figure S1. Chemical fingerprint of raw herb extract of DS (**A**) and SQ (**B**) analysed by UPLC-PDA. (1) NR1, (2) Rg1, (3) Rb1, (4) Rg2, (5) Rd, (6) DSS, (7) SB, (8) SA, (9) DT, (10) CT, (11) T1, and (12) TIIA [40].

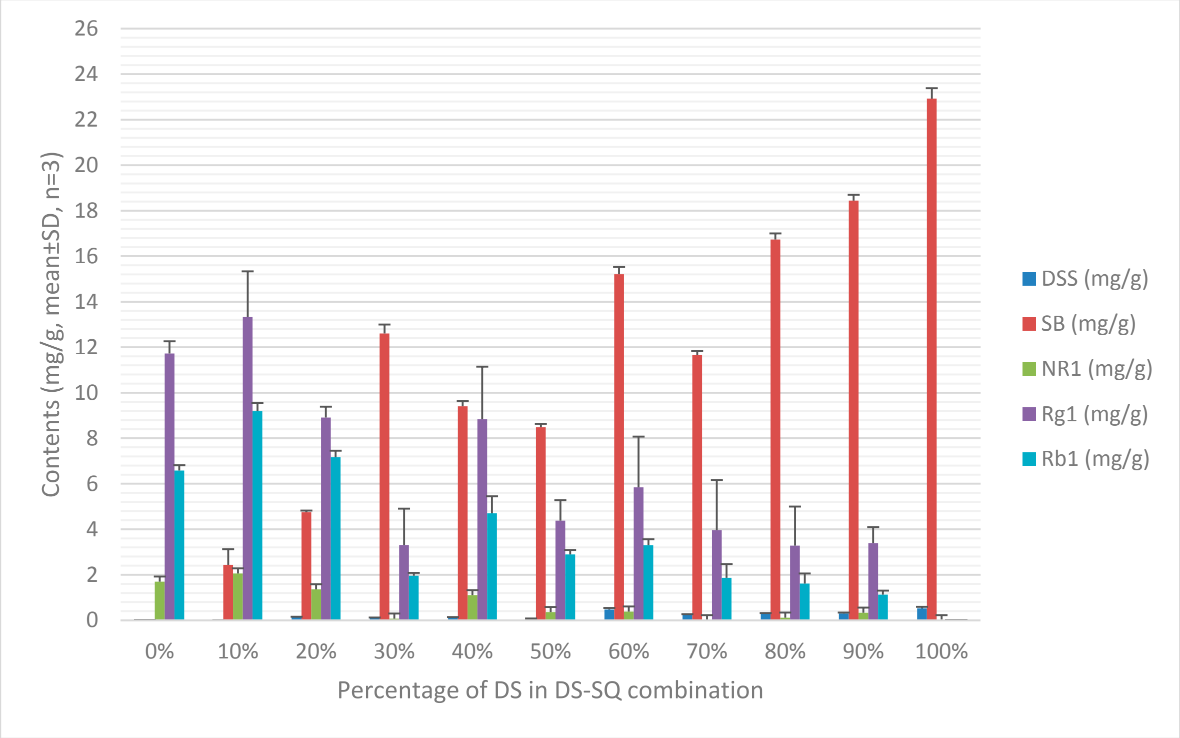

Figure S2. Contents (mg/g, mean±SD, *n* = 3) of DSS, SB, NR1, Rg1, and Rb1 in DS-SQ combinations extract by UPLC-PDA.
